# Twin-Twin Transfusion Syndrome with Anemia-Polycythemia: Prevalence, Characteristics, and Outcome

**DOI:** 10.3390/jcm8081129

**Published:** 2019-07-30

**Authors:** Lisanne S. A. Tollenaar, Femke Slaghekke, Jeanine M. M. van Klink, Sophie G. Groene, Johanna M. Middeldorp, Monique C. Haak, Frans J. C. M. Klumper, Dick Oepkes, Enrico Lopriore

**Affiliations:** 1 Division of Fetal Medicine, Department of Obstetrics, Leiden University Medical Center, 2333 ZA Leiden, The Netherlands; 2 Division of Neonatology, Department of Pediatrics, Leiden University Medical Center, 2333 ZA Leiden, The Netherlands

**Keywords:** monochorionic twin, twin-twin transfusion syndrome, twin anemia polycythemia sequence, survival, neurodevelopmental outcome, laser surgery

## Abstract

The aim of this study was to estimate the prevalence of co-existing anemia-polycythemia (AP) in twin pregnancies with twin-twin transfusion syndrome (TTTS) prior to laser surgery, and to evaluate the characteristics and outcomes in TTTS twins with and without AP. All TTTS cases treated with laser between 2001 and 2019 were retrospectively reviewed for the presence of AP before surgery. AP was defined as delta middle cerebral artery–peak systolic velocity > 0.5 multiples of the median. The primary outcome was a composite of perinatal survival and severe neurodevelopmental impairment (NDI). Secondary outcomes included procedure-related characteristics, severe neonatal morbidity, and disease-free survival. In total, 66% (461/696) of TTTS twin pregnancies were eligible for analysis. AP was detected in 15% (70/461) of the TTTS twins prior to laser surgery. Gestational age at laser was higher in the TTTS+AP group compared to the TTTS-only group—21.0 weeks (interquartile rage (IQR): 18.8–24.0) versus 19.3 weeks (IQR: 17.3–21.9), respectively (*p* < 0.0001). Fewer placental anastomoses were detected in the TTTS+AP group than in the TTTS-only group—five (IQR: 4–6) versus six (IQR: 5–8), respectively (*p* < 0.0001). Perinatal survival was 77% (599/782) in the TTTS-only group and 83% (118/142) in the TTTS+AP group (*p* = 0.130). Severe NDI was 8% (28/370) in TTTS-only and 3% (2/74) in TTTS+AP. TTTS-only twins showed more severe neonatal morbidity than twins with TTTS+AP—23% (132/575) versus 11% (13/115), respectively (*p* = 0.005). Disease-free survival was lower in the TTTS-only group compared to the TTTS+AP group—62% (341/548) versus 73% (72/98), respectively (*p* = 0.046). Thus, AP complicates 15% of TTTS twins prior to laser. TTTS+AP twins show a different placental angioarchitecture, a later time of onset of the disease, and a more favorable outcome.

## 1. Introduction

Monochorionic twins share one placenta and have their blood circulation connected to each other via placental anastomoses, which allow blood to transfer bidirectionally between the two fetuses. Unbalanced feto-fetal blood flow may lead to twin-twin transfusion syndrome (TTTS) or twin anemia-polycythemia sequence (TAPS). TTTS occurs in 10% of monochorionic twin pregnancies and arises from an unbalanced net blood flow from donor to recipient through various large placental anastomoses, resulting in large amniotic fluid discordances between donor and recipient [1]. The antenatal diagnosis of TTTS is based on the presence of twin oligohydramnios-polyhydramnios sequence (TOPS) detected through ultrasound. TAPS arises from an unbalanced and chronic net transfusion through only a few minuscule (diameter < 1 mm) vascular anastomoses, resulting in large inter-twin hemoglobin differences, without the development of TOPS [2]. TAPS may occur spontaneously in 2–5% of monochorionic twins or can develop in 1–16% of the TTTS twins treated with laser surgery (post-laser TAPS) [3,4,5,6]. The antenatal diagnosis of TAPS is reached via ultrasound Doppler, showing a large discrepancy in middle cerebral artery–peak systolic velocity (MCA-PSV) [7].

Although TTTS and TAPS have been described as two separate entities, two recent reports show that a small percentage of TTTS pregnancies are diagnosed with co-existing anemia-polycythemia (AP) [8,9]. However, in both studies, the size of the group of twins with TTTS and AP was limited, hampering adequate statistical analysis with respect to clinical outcome. In this study, we sought to evaluate the prevalence of preoperative AP in a large population of TTTS pregnancies, in order to provide more reliable information regarding placental characteristics and the short- and long-term outcomes of this selective subgroup of TTTS.

## 2. Materials and Methods

In this retrospective study, all consecutive monochorionic twin pregnancies receiving laser surgery for TTTS between 2001 and 2019 were reviewed for the preoperative presence of AP. TTTS twins with complete MCA-PSV measurements at least a week prior to fetoscopic laser surgery were considered eligible for analysis. Reasons for exclusion were incomplete or lacking MCA-PSV records, triplet pregnancies with TTTS, and TTTS pregnancies with co-existing anemia due to other causes, such as red blood cell alloimmunization.

TTTS was diagnosed using the Eurofoetus criteria [10]. MCA-PSV was assessed in agreement with the previously described technique by Mari et al. [11]. The presence of AP was defined as a delta MCA-PSV > 0.5 multiples of the median (MoM), in accordance with the new antenatal classification system for TAPS. To compare the results of this study with previously published studies, a distinction was made between twins with a delta MCA-PSV > 0.5 MoM (with normal values in the donor or recipient) and the old criteria for TAPS: MCA-PSV value > 1.5 MoM in the donor and < 1.0 MoM in the recipient. MCA-PSV values were converted from Vmax into MoMs, according to the reference ranges for monochorionic diamniotic twin pregnancies published by Klaritsch et al. [12].

The following maternal, placental, neonatal, and long-term outcome data were retrospectively obtained from digital medical records: maternal age, gravidity, parity, Quintero stage, gestational age at laser, gestational age at birth, sex, birth-weight discordance, number and type of anastomoses at fetoscopy, residual anastomoses, recurrent TTTS, recurrent TTTS with AP, post-laser TAPS, perinatal survival, severe neonatal morbidity, and severe neurodevelopmental impairment (NDI). The number of anastomoses on the placenta was counted by the operating surgeon during fetoscopic laser surgery. Severe neonatal morbidity was defined as the presence of at least one of the following conditions within 28 days after birth: respiratory distress syndrome requiring mechanical ventilation or surfactant, necrotizing enterocolitis stage 2 or higher [13], patent ductus arteriosus requiring medical therapy or surgical closure, and severe cerebral injury (at least one of the following: intraventricular hemorrhage grade 3 or higher [14], cystic periventricular leukomalacia grade 2 or higher [15], ventricular dilatation > 97th percentile [16], arterial or venous infarct, or porencephalic or parenchymal cysts). Neurodevelopment was assessed at two years of age using the Bayley Scales of Infant and Toddler Development second and third edition (Bayley-II and III), according to the standard care after fetal therapy in our center. Severe NDI was defined as at least one of the following: severe cognitive or motor delay (IQ score < 70 (−2 standard deviations (SD)), bilateral blindness, bilateral deafness (requiring amplification), or cerebral palsy (Gross Motor Function Classification System (GMFCS) ≥ stage two). The severity of cerebral palsy was classified according to the GMFCS for Cerebral Palsy [17].

The primary outcomes were perinatal survival and severe NDI. Secondary outcomes included procedure-related characteristics (gestational age at laser, coagulated placental anastomoses, occurrence of post-laser TAPS, and recurrent TTTS) severe neonatal morbidity, and disease-free survival. Disease-free survival was defined as survival without severe NDI. Outcomes were compared between twins with TTTS (TTTS-only) and twins with TTTS and AP (TTTS+AP).

Statistical analyses were performed using SPSS version 23.0 (IBM, Armonk, NY, USA). Data are reported as medians and interquartile ranges (IQR). The Mann–Whitney U test and the chi-squared test were used to calculate differences for continuous and categorical variables, respectively. To account for the fact that observations between co-twins are not independent, the generalized estimating equation module was performed for analyses per fetus or neonate. A *p*-value < 0.05 was considered to indicate statistical significance.

## 3. Results

**Figure 1:**
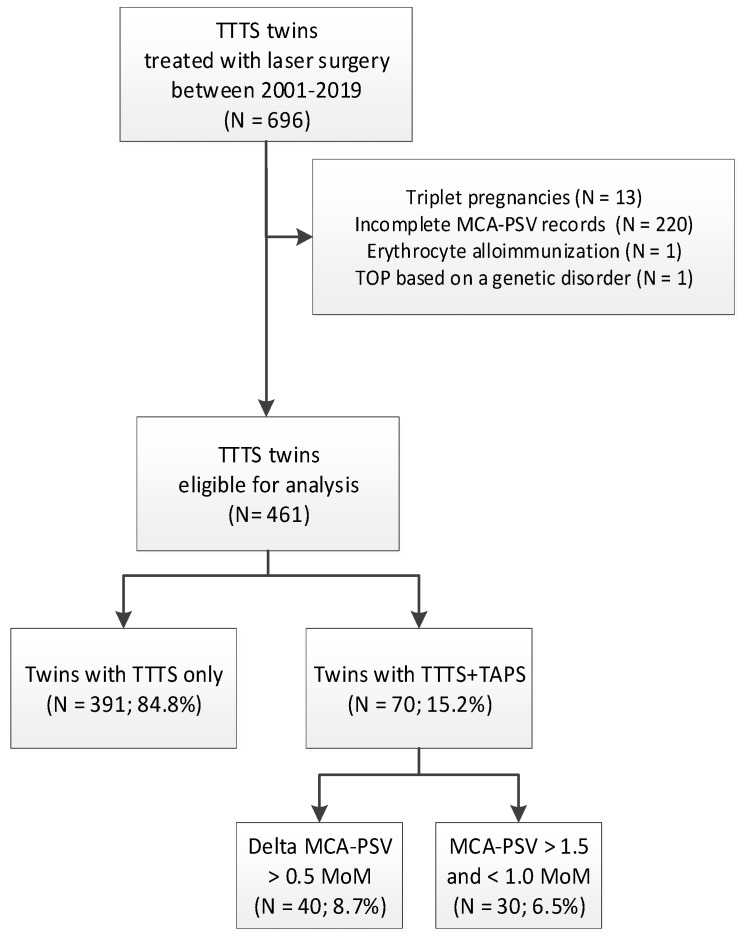
Flowchart of the derivation of the study population. TTTS: twin-twin transfusion syndrome; MCA-PSV: middle cerebral artery–peak systolic velocity; TAPS: twin anemia polycythemia sequence; MoM: multiples of the median.

Between 2001 and 2019, a total of 696 monochorionic twins with TTTS were treated with laser. Details on the derivation of the study population are shown in Figure 1. In total, 235 cases were excluded due to being a triplet (*N* = 13), incomplete MCA-PSV records prior to laser (*N* = 220), erythrocyte alloimmunization (*N* = 1), and TOP based on a genetic disorder (*N* = 1). Out of the 461 TTTS twins that were eligible for analysis, 391 twins (85%) were diagnosed with TTTS-only and 70 TTTS twins (15%) presented with AP before laser surgery. Of the 70 TTTS+AP twins, 30 cases (6.5%) had an MCA-PSV value > 1.5 MoM in the donor and < 1.0 MoM in the recipient, and 40 cases (8.7%) showed a delta MCA-PSV > 0.5 MoM, with a normal MCA-PSV value in either the donor or recipient.

Baseline characteristics of the population are presented in Table 1. Median maternal age, gravidity, parity, and Quintero stage were similar for both groups. In Table 2, procedure-related characteristics are shown. Compared to twins with TTTS only, TTTS+AP twins were more likely to receive laser treatment at a higher gestational age (*p* < 0.0001), and showed a lower total number of placental anastomoses prior to intervention (*p* < 0.0001). All types of anastomoses (arterio-venous (AV), veno-arterial (VA), arterio-arterial (AA), and veno-venous (VV)) were less frequently present in twins with TTTS+AP, although the difference in the total number of AA anastomoses failed to reach statistical significance. There was no difference in the development of recurrent TTTS or post-laser TAPS.

Table 3 depicts characteristics on short- and long-term outcome for twins with TTTS only and twins with TTTS+AP. Twins with TTTS only were delivered at a similar gestational age when compared to twins with TTTS+AP—33.0 (IQR 29.2–35.6) versus 33.1 (IQR 29.9–35.6), respectively (*p* = 0.556). Perinatal survival was 77% (599/782) and 83% (118/142) for TTTS-only twins and for TTTS+AP twins, respectively (*p* = 0.130). Fetal demise was observed in 20% (157/182) of twins with TTTS only and 16% (22/140) of TTTS+AP twins (*p* = 0.289). Neonatal mortality occurred in 4% (26/625) of the live-born twins with TTTS only and in 2% (2/118) of TTTS+AP twins (*p* = 0.090). In total, 23% (132/575) of the twins with TTTS only had severe neonatal morbidity, as opposed to 11% (13/115) of twins with TTTS+AP (*p* = 0.005). The rate of respiratory distress syndrome differed significantly between the TTTS-only group and the TTTS+AP group—20% (114/575) versus 7% (8/115), respectively.

At the time of the study, 74% (579/787) of the study population was older than two years of age and eligible for follow-up evaluation. In total, 21% (126/579) was lost to follow-up, primarily explained by the lack of follow-up between 2006 and 2007, due to organizational issues. Follow-up was incomplete in 2% (9/579) of the group. A total of 77% (444/579) of the survivors had complete follow-up and were included in the analyses. Severe NDI was observed in 8% (28/370) of the TTTS-only survivors and in 3% (2/74) of the TTTS+AP survivors, *p* = 0.053. The rate of severe cognitive delay was significantly higher in the TTTS-only group than in the TTTS+AP group—3% (11/370) versus 0% (0/74), respectively (*p* = 0.007). Disease-free survival differed significantly between TTTS-only and TTTS+AP survivors—62% (341/548) and 73% (72/98), respectively (*p* = 0.046).

## 4. Discussion

This study showed that 15% of the TTTS twins presented with co-existing anemia in the donor and polycythemia in the recipient prior to fetoscopic laser surgery. Twins with TTTS+AP had a later gestational age at laser, fewer placental anastomoses, and were characterized by a more favorable short- and long-term outcome compared to twins with TTTS-only. Since TTTS+AP twins differed on multiple levels from TTTS-only, an alternative name to distinguish between the two types might be indicated. We therefore proposed the suffix ‘AP’ for TTTS cases that present with delta MCA-PSV > 0.5 MoM.

We reported the prevalence of co-existing AP in TTTS pregnancies to be much higher compared to the studies previously conducted by Van Winden et al. and Donepudi et al., which showed a prevalence of AP of 2.4% and 8.3% in 369 and 133 TTTS twins, respectively [8,9]. The higher detection rate in our study is likely to be attributed to the use of the new antenatal criterion for TAPS: delta MCA-PSV > 0.5 MoM. This choice was based on a recently published study from our group, which showed that delta MCA-PSV > 0.5 MoM was a superior predictor for TAPS compared to the MCA-PSV cut-off levels of >1.5 MoM and <1.0 MoM [7]. Based on the old criterion, the prevalence of AP (6.5%) was roughly comparable to the findings of Donepudi et al. [8].

We found no difference in maternal baseline characteristics between twins with TTTS only and TTTS+AP twins. In contrast, Van Winden et al. demonstrated that twins with TTTS+AP were less likely to be multiparous [9], whereas Donepudi et al. showed that mothers of TTTS+AP twins had an increased chance of being multiparous and older [8]. Given the limited number of TTTS+AP cases in both studies (*N* = 11 and *N* = 9, respectively), these findings are probably due to coincidence, since it is unlikely for these factors to have a causal relationship with the pathogenesis of TTTS or TAPS.

Remarkably, twins with TTTS only showed a higher prevalence of severe neonatal morbidity than twins with TTTS+AP. This finding can be primarily explained by the large difference in respiratory distress syndrome between the two groups. Although strict statistical significance is lacking, there also seems to be a modest trend towards an increased risk for patent ductus arteriosus, severe cerebral injury, and neonatal mortality in twins with TTTS only. Notably, adverse short-term outcomes seem to be translated to an impaired long-term outcome as well; twins with TTTS only had a higher incidence of long-term NDI, almost reaching statistical significance. In addition, the rate of severe cognitive delay was significantly lower in twins with TTTS+AP. Moreover, disease-free survival was significantly higher in twins with TTTS+AP than in twins with TTTS only. The reason for the discrepancy in outcome between the two groups is not clear and has not been reported in the studies from Donepudi et al. and Van Winden et al. [8,9]. Since Quintero stage and gestational age at birth were comparable, those factors are not likely to be of influence and other explanations need to be envisaged. Since twins with TTTS only received laser therapy at a significantly lower gestational age, the time of onset of the disease might play a role in perinatal outcome. Possibly, fetal development is more prone to the detrimental effects of TTTS earlier in pregnancy. Further investigation into the role of time of onset of TTTS on perinatal outcome is needed to explore this hypothesis.

Although TTTS and TAPS have been described as two mutually different entities, the subset of TTTS+AP twins in our study seems to suggest that these two feto-fetal transfusion disorders might be part of a spectrum of findings, with a certain degree of overlap between the two. Illustratively, TTTS+AP twins resembled spontaneous TAPS twins in various ways. Confirming findings from previous studies, twins with TTTS+AP displayed a significantly lower total number of anastomoses at the placental surface [8,9]. This lower number of anastomoses was mainly explained by less AV and VA anastomoses, although AA and VV anastomoses were also less frequently present in placentas from twins with TTTS+AP. TAPS placentas are known to have only a few minuscule (<1 mm) vascular anastomoses, and usually do not show AA and/or VV anastomoses [18,19,20,21]. Furthermore, TTTS+AP twins were more likely to receive laser therapy at a higher gestational age, which might reflect a later time of onset of the disease. This finding is in agreement with the time of onset of spontaneous TAPS, which tends to develop later in pregnancy, whereas TTTS is mostly detected early in the second trimester [22].

A principle limitation of this study is the inability to measure the diameter of the anastomoses during laser treatment. In the last decade, knowledge with respect to the pathophysiology of TTTS and TAPS has increased significantly as result of postpartum macroscopic examination of the vascular anastomoses on monochorionic placentas injected with color dye. However, this retrospective study was only carried out in TTTS+AP twins treated with laser coagulation, thereby hampering any quantification of the original placental anastomoses postnatally. Notably, Donepudi et al. did report on the size of the anastomoses in TTTS+AP pregnancies, classifying them into typical or non-typical TAPS anastomoses, but these findings were solely based on a visual inspection of the vascular equator during fetoscopy and not on quantified measurements [8]. Studies with non-lasered placentas from TTTS+AP twins might help to further unveil the pathophysiological mechanism behind this subgroup of TTTS twins, and more importantly, might offer more insight into the role of anastomoses in the entire spectrum of feto-fetal transfusion disorders.

In conclusion, co-existing AP was detected in 15% of the TTTS twins prior to laser surgery. This study shows that, compared to twins with TTTS only, twins with TTTS+AP show a different placental angioarchitecture, a later time of onset of the diseases and a more favorable short- and long-term outcome. Since TTTS+AP twins differ on multiple levels from twins with TTTS only, we proposed the use of the suffix ‘AP’ for TTTS cases that present with delta MCA-PSV > 0.5 MoM antenatally. Adequate distinction between the two types of TTTS can only be made when MCA-PSV measures are routinely taken throughout pregnancy.

## Figures and Tables

**Table 1:** Baseline characteristics for twins with TTTS only and TTTS+AP (anemia-polycythemia) twins.

	TTTS-only (*N* = 391 Pregnancies, *n* = 782 Fetuses)	TTTS+AP (*N* = 70 Pregnancies *n* = 140 Fetuses)	*p*-Value
Maternal age (years)	32 (28–35)	30 (27–34)	0.438
Gravidity	2 (1–3)	2 (1–3)	0.380
Parity	1 (0–1)	1 (0–1)	0.778
Male	189/388 (49)	37/69 (54)	0.552
Cesarean	286/776 (37)	58/140 (41)	0.423
Delta MCA-PSV (MoM)	0.2 (0.1–0.3)	0.7 (0.6–0.9)	**<0.0001**
Quintero stage			0.198
I	58/391 (15)	11/70 (16)	
II	140/391 (36)	16/70 (23)	
III	180/391 (46)	40/70 (57)	
IV	13/391 (3)	3/70 (4)	

Data are median (IQR) or n/N (%). TTTS: twin-twin transfusion syndrome; TAPS: twin anemia polycythemia sequence; MCA-PSV: middle cerebral artery–peak systolic velocity; MoM: multiples of the median. Bold indicates statistical significance.

**Table 2:** Procedure-related characteristics for twins with TTTS-only and twins with TTTS+AP.

	TTTS-only (*N* = 391 Pregnancies, *n* = 782 Fetuses)	TTTS+AP (*N* = 70 Pregnancies *n* = 140 Fetuses)	*p*-Value
Gestational age at laser	19.3 (17.3–21.9)	21.0 (18.8–24.0)	**<0.0001**
Total number of anastomoses on fetoscopy	6 (5–8)	5 (4–6)	**<0.0001**
Number of AV-anastomoses	3 (3–5)	3 (2–4)	**0.018**
Number of VA-anastomoses	2 (1–3)	2 (1–3)	**0.012**
Presence of AA-anastomoses	54/371 (15)	5/67 (7)	0.118
Presence of VV-anastomoses	35/371 (9)	0/67 (0)	**0.009**
Presence of residual anastomoses	53/277 (19)	9/50 (18)	0.742
Recurrent TTTS	3/291 (1)	1/70 (1)	0.458
Recurrent TTTS with AP	2/391 (1)	0/70 (0)	0.549
Post-laser TAPS	37/387 (10)	6/70 (9)	0.855

Data are median (IQR) or n/N (%), AV: arterio-venous; VA: veno-arterial; AA: arterio-arterial; VV: veno-venous; TTTS: twin-twin transfusion syndrome; AP: anemia-polycythemia; TAPS: twin anemia polycythemia sequence. Bold indicates statistical significance.

**Table 3:** Perinatal outcome for twins with TTTS-only and TTTS+AP twins.

	TTTS-only (*N* = 391 Pregnancies, *n* = 782 Fetuses)	TTTS+AP (*N* = 70 Pregnancies *n* = 140 Fetuses)	*p*-Value
Gestational age at birth (weeks)	33.0 (29.2–35.6)	33.1 (29.9–35.6)	0.556
Birth-weight discordance (%)	11.5 (4.9–21.3)	10.8 (4.6–20.8)	0.953
Perinatal survival	599/782 (77)	118/142 (83)	0.130
Fetal demise	157/782 (20)	22/140 (16)	0.283
Neonatal mortality	26/625 (4)	2/118 (2)	0.090
Severe neonatal morbidity	132/575 (23)	13/115 (11)	**0.005**
Respiratory distress syndrome	114/575 (20)	8/115 (7)	**<0.0001**
Patent ductus arteriosus	19/575 (3)	1/115 (1)	0.054
Necrotizing enterocolitis	17/575 (3)	3/115 (3)	0.873
Severe cerebral injury	33/575 (6)	3/115 (3)	0.082
Severe NDI	28/370 (8)	2/74 (3)	0.053
Severe cognitive delay	11/370 (3)	0/74 (0)	**0.007**
Severe motor delay	14/370 (4)	2/74 (3)	0.605
Bilateral blindness	0/370 (0)	0/74 (0)	1.000
Bilateral deafness	4/370 (1)	0/74 (0)	0.177
Cerebral palsy	12/370 (3)	2/74 (3)	0.682
Disease-free survival	341/548 (62)	72/98 (73)	**0.046**

Data are median (IQR) or n/N (%). TTTS: twin-twin transfusion syndrome; TAPS: twin anemia polycythemia sequence; NDI: neurodevelopmental impairment. Bold indicates statistical significance.
